# Injuries at a Canadian National Taekwondo Championships: a prospective study

**DOI:** 10.1186/1471-2474-5-22

**Published:** 2004-07-27

**Authors:** Mohsen Kazemi, Willy Pieter

**Affiliations:** 1 Department of Clinical Studies, Canadian Memorial Chiropractic College, Toronto, Ontario, Canada; 2 School of Health Sciences, Science University of Malaysia, Kelantan, Malaysia

## Abstract

**Background:**

The purpose of this prospective study was to assess the injury rates in male and female adult Canadian Taekwondo athletes relative to total number of injuries, type and body part injured.

**Methods:**

Subjects (219 males, 99 females) participated in the 1997 Canadian National Taekwondo Championships in Toronto, Canada. Injuries were recorded on an injury form to documents any injury seen and treatment provided by the health care team. These data were later used for this study. The injury form describes the athlete and nature, site, severity and mechanism of the injury.

**Results:**

The overall rate of injuries was 62.9/1,000 athlete-exposures (A-E). The males (79.9/1,000 A-E) sustained significantly more injuries than the females (25.3/1,000 A-E). The lower extremities were the most commonly injured body region in the men (32.0 /1,000 A-E), followed by the head and neck (18.3/1,000 A-E). Injuries to the spine (neck, upper back, low back and coccyx) were the third most often injured body region in males (13.8/1,000 A-E). All injuries to the women were sustained to the lower extremities. The most common type of injury in women was the contusion (15.2/1,000 A-E). However, men's most common type of injury was the sprain (22.8/1,000 A-E) followed by joint dysfunction (13.7/1,000A-E). Concussions were only reported in males (6.9/1,000 A-E). Compared to international counterparts, the Canadian men and women recorded lower total injury rates. However, the males incurred more cerebral concussions than their American colleagues (4.7/1,000 A-E).

**Conclusions:**

Similar to what was found in previous studies, the current investigation seems to suggest that areas of particular concern for preventive measures involve the head and neck as well as the lower extremities. This is the first paper to identify spinal joint dysfunction.

## Background

With the inclusion of Taekwondo as a medal sport in the 2000 Olympic Games interest has gained momentum from participants, national governments and scientists alike. With increased participation, the issue of safety becomes very important and timely. The rules and regulations with regards to these Championships were that of the World Taekwondo Federation [1]. All athletes were sixteen years of age and older and held black belt first degree as a minimum requirement to compete. At the time of data collection, punches were allowed to the front of the torso in the area covered by the chest protector worn by the athletes. No punches were allowed to the head or other parts of the body. Kicks were allowed to the torso and head, the latter of which was covered by a helmet similar to the one worn in amateur boxing. Regardless of the area of contact, only one point was granted by the referees for a successful blow. One could win the match by means of a knockout, therefore, contact was encouraged. There was a change of rules introduced in 2003, which included granting two points for head shots and an additional point for an eight-count knockout [1].

Prospective studies on Taekwondo injuries sustained at single tournaments have been conducted before [1-3]. For instance, Zemper and Pieter [2] found injury rates for American elite male Taekwondo athletes to be 127.4/1,000 athlete-exposures and for females, 90.1/1,000 athlete-exposures. One athlete-exposure (A-E) refers to one athlete being exposed to the possibility of being injured. Since there are always two athletes competing during any one bout, there are two athlete-exposures per bout [2,3]. In a later study, Pieter et al. [3] reported injury rates of 139.5/1,000 A-E and 96.5/1,000 A-E for European men and women, respectively. In neither study were the differences tested for statistical significance. However, at a recreational tournament in the United Kingdom, the men (51.3/1,000 A-E) sustained statistically significantly more injuries than the women (47.6/1,000 A-E) [4].

It is not clear why the men in the studies mentioned above sustained more injuries. Sample size may be a factor. For instance, in a prospective study covering multiple Taekwondo tournaments, Pieter and Zemper [5] reported a statistically significantly higher injury rate for the women (105.5/1,000 A-E versus 95.1/1,000). In the largest prospective judo injury study to date, young and adult females (130.6/1,000 A-E) incurred a higher injury rate than their male counterparts (122.6/1,000 A-E) [6]. In addition to sample size, the number of female competitors is also lower [5,6].

As expected in a collision sport, the contusion was the most frequently occurring injury type in both male and female Taekwondo athletes [2-4]. At the elite level, more serious injuries such as fractures and cerebral concussions also occur [3,7]. Men seem to incur more of these serious injuries [2,3]. Less information is available in karate, for the authors have not consistently reported the injuries by gender. Based on what is known from prospective studies on elite karate athletes, the men also seem to incur more serious injuries, some of which have led to time loss [8,9].

The body region most frequently affected in single tournaments involving both recreational and elite Taekwondo athletes is the lower extremities, especially the (instep of the) foot [2-4]. Since full-contact or Olympic Taekwondo is characterized by kicking, this should come as no surprise.

There is a lack of data on gender differences in injuries to body region and body part in karate. Most prospective studies combined the injury rates for males and females [e.g., [8]]. Using a prospective design, Pieter [10] reported that the head and neck sustained most of the injuries in both elite male and female karate athletes.

There is also scarce information on male-female comparisons on body regions injured in judo athletes. Our own studies in judo seem to indicate that in the women, the upper extremities are mostly affected, while in the men, the head and neck as well as the lower extremities are injured most often [11,12].

In line with the frequent use of the legs in Taekwondo, the main injury mechanism was found to be delivering or receiving a kick [2,5]. Further analysis revealed that the roundhouse kick was most often implicated, especially in men [3,4,13]. The fact that injuries occur as a result of receiving a kick may be partially related to unblocked attacks, which has led to the recommendation for the coaches to work on improving the blocking skills or evasive maneuvers of their athletes [2].

The purpose of this study was to identify and compare the rates of injury in Canadian male and female Taekwondo competitors relative to total number of injuries, type, body part injured and mechanism.

## Methods

**Figure 1:**
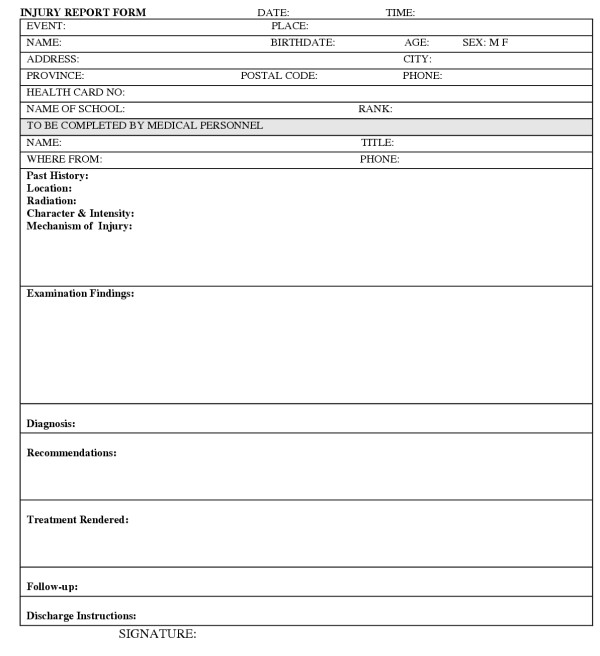
Injury data collection form.

Subjects (219 males and 99 females) participated in the 1997 Canadian National Taekwondo Championships in Toronto, Canada. Injuries were recorded on an injury form to document any injury seen and treatment provided by the health care team as it was required by law. The first author was the only person who kept the injury forms and entered the data, therefore, keeping the identity of athletes confidential. Oral consent was obtained from the athletes for assessment and providing therapy. Data describe the athlete and nature, site, severity and mechanism of the injury. No reliability and validity information for the instrument is available and this study was carried out to pre-test the injury data collection form (Figure 1).

Injuries were diagnosed by the tournament physician (MK), who has been the national team chiropractic physician for several years and is an experienced (black belt) Taekwondo athlete himself. One injury form was filled out by the attending physician for each time the athlete reported a new injury. However, at each presentation there could be more than one injury reported on the same Injury Report form. For the purposes of this study, an athlete was considered injured if any of the following conditions applied [14]: 1) any circumstance that forced the Taekwondo athlete to leave the competition; 2) any circumstance for which the referee or athlete had to stop competition; 3) any circumstance for which the athlete requested medical attention. In other words, the definition included so-called time-loss injuries (stoppage of a bout) as used in the NCAA Injury Surveillance System [15].

Injury rates were calculated from matches fought using the basic rate formula: (# injuries / # athlete-exposures) × 1,000 = # injuries per 1,000 athlete-exposures (A-E). The Colorado concussion classification was utilized in management of the concussions [16,17]. According to this classification, a first degree concussion is identified by confusion, no loss of memory and no loss of consciousness (LOC). A second degree involves confusion, loss of memory but no LOC and the third degree is when there is LOC [16,17].

## Results

The age range for the males was 17–34 years with a mean of 24.2 years and for the females, 16–26 years with a mean of 21 years. The age was not recorded for 3 males and 1 female. Table 1 displays the injury data and rates for the Canadian Taekwondo athletes.

The lower extremities were the most commonly injured body region in the men (32.0 /1,000 A-E), followed by the face (eyes, nose, cheek, lips, jaw; 18.3/1,000 A-E), and the spine (neck, upper back, low back and coccyx) (13.8/1,000 A-E). If the head and neck (which includes the face area) are combined, as was done in other studies [4,7], this body region incurred the second highest injury rate: 24.9/1,000 A-E. All injuries to the women were sustained to the lower extremities with the foot incurring most of the injuries (15.2/1,000 A-E; Table 2).

The top five injuries in the males include the sprain (22.8/1,000 A-E), followed by the joint dysfunction (13.7/1,000 A-E), contusion and laceration (11.4/1,000 A-E each), and strain (9.1/1,000 A-E). The cerebral concussion is ranked sixth (6.9/1,000 A-E). There were one third degree and two first degree concussions. In the women, the contusion was the most often occurring injury (15.2/1,000 A-E), followed by the sprain and strain (5.1/1,000 A-E each; Table 3).

Table 4 displays the rates of the injury mechanisms by gender. Receiving a kick by the men included those connecting with the head/face (18.3/1,000 A-E), trunk (6.9/1,000 A-E), and thigh (2.3/1,000 A-E). Delivering a kick as an injury mechanism in the men included, among others, kicking to the elbow (6.9/1,000 A-E), kicking with the toes, to the trunk and with the knee (2.3/1,000 A-E each). In the women, delivering a kick (10.1/1,000 A-E) was the main injury mechanism and comprised kicks to the elbow, while the kick that was received involved a knee kick.

## Discussion

The injuries incurred by the Canadian Taekwondo athletes compare favourably to those found by others. As mentioned above, American elite athletes recorded injury rates of 127.4/1,000 A-E (men) and 90.1/1,000 A-E (women) [2], while European colleagues had rates of 139.5/1,000 A-E (men) and 96.5/1,000 A-E (women) [3]. At one Greek national championship, the men (20.6/1,000 A-E) sustained statistically significantly fewer injuries than the women (36.4/1,000 A-E) [13]. The total injury rate of the Canadian male and female Taekwondo athletes combined, was also lower than that of their African counterparts (86.6/1,000 A-E) [7]. At an Open British tournament, injury rates of 51.3/1,000 A-E (men) and 47.6/1,000 A-E (women) were reported [4]. However, the athletes competing at this particular tournament were of sub-elite level. It is hypothesised that injuries may be related to level of skill and experience, although confirmatory research still needs to be carried out [18]. All competitors were black belts, but no information is available on their experience in Taekwondo and in competition. Future research should include general Taekwondo as well as competition-specific experience in addition to belt level. Comparative data gleaned from prospective studies on other martial arts injuries incurred at single tournaments are depicted in table 5.

In view of the nature of the sport, as alluded to above, it is not surprising to find the lower extremities to sustain most of the injuries as was found in previous studies as well [2,3,13]. Within the lower extremities, the foot (i.e., instep) was the most often injured body part, as was the case with the females in the present study, which led to the suggestion for the Taekwondo governing bodies to recommend padding to help decrease injuries to this site [22]. In karate, on the other hand, the head and neck incur most of the injuries [8,10], while in judo, the upper extremities are more at risk [6,11].

As expected, the contusion was found to be the most frequently occurring injury type in other studies on Taekwondo injuries [e.g., [2,3,13]]. The sprain ranked in the top three of most frequently occurring injuries across several tournaments [5]. The contusion was also the most often occurring injury in karate [9,10], while the epistaxis ranked second in Dutch men and women [10]. In Finnish elite male karate athletes the laceration was ranked second and the epistaxis in women [9]. In judo, the strain in men and the abrasion in women were sustained most often [11,12]. Since the sample sizes in the current investigation as well as in the aforementioned karate and judo studies are rather small, more research is needed to arrive at more definitive conclusions. However, differences between the martial arts in terms of techniques used and competition rules undoubtedly play a major role.

Of more concern, however, is the occurrence of cerebral concussions. The Canadian males recorded a higher rate than found in American (4.7/1,000 A-E) [2] and Greek (1.0/1,000 A-E) [13] elite Taekwondo athletes, but lower than those competing in the 1993 European Cup (15.5/1,000 A-E) [3] and the 1991 World Championships (15.3/1,000 A-E) [23]. The Canadians also recorded lower rates than elite Dutch karate athletes (13.2/1,000 A-E) competing under semi-contact rules [10]. Given the serious implications of these injuries, preventive measures, testing of equipment and follow-up research are urgently needed [e.g., [18]].

In accordance with what was found previously, the injury mechanisms included both receiving and delivering kicks for men and women alike [2,5]. The men, more than the women, tended to get injured as a result of receiving a kick [2,3]. It is suggested that the technique most likely implicated is the roundhouse kick [3,4,13]. Kicking the elbow typically leads to injury, especially if the kick is executed with the instep of the foot, such as when using the roundhouse kick to attack or counter-attack. Yet another reason to implement foot padding, as already mentioned above. In karate, punching is the main injury mechanism for both men and women [8,10], which may be related to the head and neck region being most frequently injured.

More research on Canadian Taekwondo athletes of different age groups and skill levels is needed. Age in the present study is not believed to have played a role in the injuries sustained. The injury profile of the Canadians is quite similar to those found in other studies with Taekwondo athletes in their early twenties [2,3,5]. Children and juniors in Taekwondo were reported as incurring higher injury rates than adults [4,13]. Future studies should also include time lost due to injury.

Joint dysfunction was identified as the second most common injury sustained by male athletes (13.7/1,000 A-E). Haldeman [24] defines joint dysfunction quoting Drum (1973) as,"Joint mechanics showing area disturbances of function without structural change; subtle joint dysfunctions affecting quality and range of joint motion. They are diagnosed with the aid of motion palpation, as well as stress and motion radiography investigation" [p. 623]. Greenman [25] states: "Joint dysfunction is characterized by findings of misalignment, relative fixation, loss of normal range-of-motion and end-play, tenderness, and tissue texture abnormality" [p. 13–14]. Although controversial, the term has been used widely in the literature, mostly by chiropractors, physical therapists and occasionally by biomechanists and medical doctors [24-33]. Further studies are required to validate the current finding.

## Conclusion

The total injury rates for the Canadian Taekwondo athletes compare favourably to those reported in the literature, which is contrary to what was expected based on such a small sample size. It is hypothesized that recent rule changes may have contributed to these relatively low rates when compared to those found for other single tournaments, although more research is indicated before a definitive conclusion may be drawn. Interestingly, joint dysfunction was identified for the first time, which warrants more study. The injury data collection form should also include the technique used as a specification of the injury mechanism. General Taekwondo and competition-specific experience in addition to belt rank should also be recorded.

## Competing interest

None declared.

## Authors' contributions

MK collected the data, designed the Injury Report form, and wrote the initial draft of the manuscript. WP did the result part, revised and proof read the manuscript.

## Pre-publication history

The pre-publication history for this paper can be accessed here:



## Figures and Tables

**Table 1:** Injury rates (95%CI) in adult Canadian Taekwondo athletes.

	Men	Women	Total
Number of athletes	219	99	318
Number of reported injuries	35	5	40
Number of athlete-exposures (AE)	438	198	636
Injury rates			
-- per 100 athletes	16.0 (10.7–21.3)	5.1 (0.7–9.5)	12.6 (8.7–16.5)
-- per 1,000 AE	79.9 (53.4–106.4)	25.3 (3.2–47.4)	62.9 (43.4–82.4)

**Table 2:** Distribution of injuries by body part per 1,000 athlete-exposures.

	Men			Women	
Body part	Number	Rate	Body part	Number	Rate

Head	3	6.9	Hamstrings	1	5.1
Eyes	2	4.6	Ankle	1	5.1
Nose	3	6.9	Foot	3	15.2
Cheek	1	2.3			
Lip	1	2.3			
Jaw	1	2.3			
Neck	1	2.3			
Hands	3	6.9			
Upper back	1	2.3			
Low back	2	4.6			
Pelvis	1	2.3			
Coccyx	2	4.6			
Hamstrings	3	6.9			
Leg	2	4.6			
Ankle	5	11.4			
Foot	2	4.6			
Toes	2	4.6			
Total	35	79.9	Total	5	25.3

**Table 3:** Distribution of injuries by injury type per 1,000 athlete-exposures.

	Men			Women	
Injury type	Number	Rate	Injury type	Number	Rate

Sprain	10	22.8	Contusion	3	15.2
Joint dysfunction	6	13.7	Sprain	1	5.1
Contusion	5	11.4	Strain	1	5.1
Laceration	5	11.4			
Strain	4	9.1			
Concussion	3	6.9			
Abrasion	1	2.3			
Epistaxis	1	2.3			
Total	35	79.9	Total	5	25.3

**Table 4:** Distribution of injuries by mechanism per 1,000 athlete-exposures.

Men			Women		
Injury mechanism	No	Rate	Injury mechanism	No	Rate

Receiving a kick	12	27.4	Delivering a kick	2	10.1
Delivering a kick	7	16.0	Receiving a kick	1	5.1
Simultaneous kicks	1	2.3	Not recorded	2	10.1
Other	3	6.9			
Not recorded	12	27.4			
Total	35	79.9	Total	5	25.3

**Table 5:** Comparative injury rates per 1,000 athlete-exposures (95%CI) in adult martial arts athletes*.

Sport/Study	Men	Women
Taekwondo (this study)	79.9 (53.4–106.4)	25.3 (3.2–82.4)
Judo [12]	48.5 (18.5–78.6)	34.3 (4.2–64.3)
Judo [19]	115.1 (90.9–139.3)	--
Judo [20]	51.3 (1.0–101.6)	125.0 (107.7–142.3)
Judo [11]	25.2 (6.5–43.8)	41.3 (14.3–68.3)
Karate [10]	168.9 (144.1–193.6)	158.5 (120.0–197.1)
Karate [19]	65.5 (43.1–87.8)	--
Karate [21]	135.6 (105.9–165.3)	--
Karate [9]	157.7 (123.6–191.8)	80.4 (27.9–132.9)

*Except for our own studies, injury rates are estimated based on the information provided by the authors

## References

[B1] http://www.wtf.org.

[B2] Zemper ED, Pieter W (1989). Injury rates during the 1988 US Olympic Team Trials for Taekwondo. Br J Sports Med.

[B3] Pieter W, Van Ryssegem G, Lufting R, Heijmans J (1995). Injury situation and injury mechanism at the 1993 European Taekwondo Cup. J Hum Mov Stud.

[B4] Pieter W, Bercades LT, Heijmans J (1998). Injuries in young and adult Taekwondo athletes. Kines.

[B5] Pieter W, Zemper ED Injuries in adult American Taekwondo athletes. In Proceedings of Fifth IOC World Congress on Sport Sciences, Sydney, Australia.

[B6] Barrault D, Achou B, Sorel R (1983). Accidents et incidents survenus au cours des compétitions de judo. Symb.

[B7] Phillips JS, Frantz JM, Amosun SL, Weitz W (2001). Injury surveillance in Taekwondo and judo during physiotherapy coverage of the seventh All Africa Games. SA J Phys.

[B8] Hillman S, Dicker G, Sali A (1993). Non contact karate injuries. Aus J Sci Med Sport.

[B9] Tuominen R (1995). Injuries in national karate competitions in Finland. Scan J Med Sci Sports.

[B10] Pieter W Injuries and mechanisms of injury in karate competition. In Proceedings of 1st World Congress on Combat Sports and Martial Arts, Université de Picardie Jules Verne, Faculté de Sciences du Sport, Amiens, France.

[B11] Pieter W, Talbot C, Pinlac V, Bercades LT (2001). Injuries at the Konica Asian Judo Championships. Acta Kines Univ Tartu.

[B12] James G, Pieter W (2003). Injury rates in adult elite judoka. Biol Sport.

[B13] Beis K, Tsaklis P, Pieter W, Abatzides G (2001). Taekwondo competition injuries in Greek young and adult athletes. Eur J Sports Traumol rel res.

[B14] Lindenfeld TN, Schmitt DJ, Hendy MP, Mangine RE, Noyes FR (1994). Incidence of injury in indoor soccer. Am J Sports Med.

[B15] McKeag DB, Hough DO, Zemper ED (1993). Primary Care Sports Medicine. Dubuque, IA: Brown & Benchmark.

[B16] Colorado Medical Society. Report of the Sports Medicine Committee (1991). Guidelines for the Management of Concussion in Sports (revised).

[B17] Kelly JP, Rosenberg JH (1998). The development of guidelines for the management of concussion in sports. J Head Trauma Rehab.

[B18] Pieter W, Caine D, Caine C, Lindner K (1996). Martial arts. In Epidemiology of Sports Injuries.

[B19] Dah C, Djessou P (1989). Accidents et incidents liés au judo et au karaté au cours d'une saison sportive [1986–1987] en Côte-d'Ivoire. Cinés.

[B20] Pieter W, De Crée C Competition injuries in young and adult judo athletes. In Proceedings of The Second Annual Congress of the European College of Sport Science, Copenhagen, Denmark.

[B21] McLatchie GR (1976). Analysis of karate injuries sustained in 295 contests. Inj Brit J Acc Surg.

[B22] Pieter W, Zemper ED, Varnes JW, Gamble D, Horodyski MB (1995). Foot injuries in Taekwondo. In Proceedings of the 38th World Congress Proceedings, Gainesville: 1995; The University of Florida College of Health and Human Performance.

[B23] Pieter W, Lufting R (1994). Injuries at the 1991 Taekwondo world championships. J Sports Traumatol rel res.

[B24] Haldeman S (1992). Principles and practice of chiropractic.

[B25] Greenman PE (1996). Principles of manual medicine.

[B26] Taylor P, Tole G, Vernon H (1990). Skin rolling technique as an indicator of spinal joint dysfunction. JCCA.

[B27] Suter E, McMorland G, Herzog W, Bray R (2000). Conservative lower back treatment reduces inhibition in knee-extensor muscles: a randomized controlled trail. J Manipulative Physiol Ther.

[B28] Kokmeyer DJ, Van der Wurff P, Aufdemkampe G, Fickenscher TC (2002). The reliability of multitest regimens with sacroiliac pain provocation tests. J Manipulatice Physiol Ther.

[B29] Knustson GA (1999). Dysafferentation: a novel tern to describe the neuropathological effects of joint complex dysfunction–a look at likely mechanisms of symptom generation. J Manipulative Physiol Ther.

[B30] Suter E, McMorland G, Herzog W, Bray R (1999). Decrease in quadriceps inhibition after sacroiliac joint manipulation in patients with anterior knee pain. J Manipulative Physiol Ther.

[B31] Toussaint R, Gawlik CS, Rehder U, Ruther W (1999). Sacroiliac dysfunction in construction workers. J Manipulative Physiol Ther.

[B32] Maigne JY, Chatellier G Comparison of three manual coccydynia treatments: a pilot study. Spine.

[B33] Harrison DE, Harricon DD, Troyanovich SJ (1997). The sacroiliac joint: a review of anatomy and biomechanics with clinical implications. J Manipulative Physiol Ther.

